# Functional Roles of Homologous Recombination and Non-Homologous End Joining in DNA Damage Response and Microevolution in *Cryptococcus neoformans*

**DOI:** 10.3390/jof7070566

**Published:** 2021-07-16

**Authors:** Kwang-Woo Jung, Jong-Hyun Jung, Ha-Young Park

**Affiliations:** 1 Radiation Research Division, Advanced Radiation Technology Institute, Korea Atomic Energy Research Institute, Jeongeup-Si 56212, Jeollabuk-Do, Korea; jungjh83@kaeri.re.kr (J.-H.J.); hypark@kaeri.re.kr (H.-Y.P.); 2 Department of Radiation Science and Technology, University of Science and Technology, Daejeon 34113, Korea

**Keywords:** DNA damage, homologous recombination, non-homologous end joining, microevolution, *Cryptococcus*

## Abstract

DNA double-strand breaks (DSBs) are the most deleterious type of DNA lesions because they cause loss of genetic information if not properly repaired. In eukaryotes, homologous recombination (HR) and non-homologous end joining (NHEJ) are required for DSB repair. However, the relationship of HR and NHEJ in DNA damage stress is unknown in the radiation-resistant fungus *Cryptococcus neoformans*. In this study, we found that the expression levels of HR- and NHEJ-related genes were highly induced in a Rad53–Bdr1 pathway-dependent manner under genotoxic stress. Deletion of *RAD51*, which is one of the main components in the HR, resulted in growth under diverse types of DNA damage stress, whereas perturbations of *KU70* and *KU80*, which belong to the NHEJ system, did not affect the genotoxic stresses except when bleomycin was used for treatment. Furthermore, deletion of both *RAD51* and *KU70*/*80* renders cells susceptible to oxidative stress. Notably, we found that deletion of *RAD51* induced a hypermutator phenotype in the fluctuation assay. In contrast to the fluctuation assay, perturbation of *KU70* or *KU80* induced rapid microevolution similar to that induced by the deletion of *RAD51*. Collectively, Rad51-mediated HR and Ku70/Ku80-mediated NHEJ regulate the DNA damage response and maintain genome stability.

## 1. Introduction

Cells have evolved to contain sophisticated DNA repair systems to maintain a high fidelity of the genome. Although environmental agents, such as ultraviolet light (UV), induce detrimental DNA lesions in living cells, intracellular processes, such as replication, also generate thousands of lesions per day [1]. For example, it is estimated that approximately 70,000 DNA lesions are produced in human cells per day [2]. Most of them are DNA single-strand breaks (SSBs) and depurination/depyrimidination, whereas 8-oxoguanine and cytosine deamination also occur less frequently than SSBs [3]. Even though DNA double-strand breaks (DSBs) are the least frequent, they are more dangerous to living organisms because they result in the loss of genetic information.

Cells exploit independent DNA repair pathways to correct mutations depending on the type of the DNA lesion. Intracellular reactive oxygen species (ROS) generated during cellular respiration or treatment of exogenous DNA damage insults produce the anoxidized base product, 8-oxoguanine (7,8-dihydro-8-oxoguanine), which results in a mismatched base pairing [4]. Glycosylases detect damaged bases and remove them by cleaving the *N*-glycosidic bond between the sugar backbone and the damaged base, thereby producing an abasic site [5]. Next, apurinic/apyrimidinic (AP) endonuclease cleaves the phosphodiester backbone of the AP site to create a nick. A DNA polymerase and ligase insert the correct base and seal the nick between the phosphodiester bond, by a process called base excision repair (BER) [6]. Nucleotide excision repair is activated by the formation of bulky DNA adducts, such as pyrimidine dimers or 6,4-photoadducts, induced by UV exposure [7]. In *Saccharomyces cerevisiae*, the damaged regions are recognized by diverse factors, such as Rad14, Rad4–Rad23, and Rad7–Rad16 complexes, and unwound by helicases, such as Rad3 and Rad25, to help endonuclease access the damage site. The endonuclease excises 25–30 nucleotides containing the damaged DNA, which results in the formation of single-stranded DNA. Next, DNA polymerases resynthesize a new complementary DNA strand, and ligases seal the nicks [8,9]. In contrast to NER and BER, the mismatch repair pathway recognizes and rectifies mispaired bases caused by DNA polymerase errors after replication [10]. First, Msh2–Msh6 or Msh2–Msh3 heterodimer proteins recognize base–base mismatches and recruit Mlh1–Pms1 complexes containing endonuclease activity [11,12]. Finally, exonuclease proteins excise a mismatched strand, followed by gap filling by DNA polymerase and ligase [13,14]. To repair DSBs, cells mainly exploit two DNA repair pathways; homologous recombination (HR) and non-homologous end joining (NHEJ) pathways that are used during specific stages of the cell cycle [15]. The NHEJ repairs the DSB by directly rejoining the broken end, often inducing loss of genetic information. In the NHEJ pathway, the Ku70/Ku80 heterodimer binds DNA ends and protects them from degradation until DNA ligase IV (Dnl4) is recruited. Then, Dnl4 plays a role in the ligation of the broken end [16]. In the HR process, the evolutionarily conserved MRX (Mre11–Rad50–Xrs2) complex is recruited to the DSB site where it participates in end resection [17]. After resection, the single-stranded DNA is coated with replication protein A and bound to Rad51, which is a recombinase for strand exchange. Next, several proteins involved in the recombination, such as Rad52, Rad54, and Rad55, are recruited to the DSB site to stimulate strand exchange [18,19]. Given that HR requires an undamaged homologous template for repair, cells exploit this pathway during the late S and G2 phases. For this reason, HR preserves genomic information without loss, in contrast to the NHEJ pathway. However, several lines of evidence indicate that DNA repair pathways for DSBs do not function separately but rather together [20,21].

Paradoxically, in pathogenic fungi, genetic alterations help pathogens adapt to the host environment, such as treatment with antifungal agents [22,23,24]. The process by which phenotypic changes are obtained by genetic alterations to adapt to the circumstances is called microevolution. Recent studies have revealed that sexual and asexual reproduction contribute to genetic variation to adjust to the host and environment [25,26]. In addition to sexual and asexual reproduction, diverse types of genetic changes caused by single-nucleotide polymorphisms (SNPs) and insertions and deletions (indels) occur during mitotic growth. SNPs are mainly recognized and repaired by the mismatch repair pathway, whereas homologous recombination is involved in the production of indels.

*C. neoformans* is one of the leading human fungal pathogens responsible for meningoencephalitis. The dried yeasts and basidiospores are inhaled through the respiratory tract and disseminated in the bloodstream. They cross the blood–brain barrier (BBB) and finally reach the brain [27]. Annually, approximately 220,000 cases of cryptococcal meningitis occur globally, and 80% of them lead to death [28]. Recently, we identified a novel transcription factor, Bdr1, that controls the expression levels of DNA repair genes after radiation exposure [29]. Furthermore, we demonstrated that Rad53 and Chk1, which are downstream factors of the PI3K pathway, cooperatively and distinctly regulate the DNA damage response as well as an oxidative stress response. According to the transcriptome analyses performed in a previous study, we identified and characterized several radiation-inducible genes, such as *RIG* genes, and DNA repair genes, such as *RAD51*, regulated by the Rad53–Bdr1 pathway [29,30]. Although *KU70* and *KU80* genes have been characterized in DNA damage stress and the homologous recombination rate in the context of *C. neoformans* [31], the relationship between HR and NHEJ in the DNA repair system and microevolution in *C. neoformans* remains elusive.

In this study, we found that expression levels of both *KU70* and *KU80* genes were induced in a Rad53–Bdr1 pathway-dependent manner, similar to HR genes, and HR and NHEJ play redundant roles in DNA damage and oxidative stress responses. In particular, we demonstrated that perturbation of the Rad51-mediated HR system increased the spontaneous mutation rate via a fluctuation assay. Notably, perturbation of *RAD51* or *KU70*/*KU80* resulted in rapid microevolution. Therefore, Rad51-mediated HR and Ku70/Ku80-mediated NHEJ cooperatively regulate the DNA damage response and control microevolution in *C. neoformans*.

## 2. Materials and Methods

### 2.1. Strains and Growth Condition

*C. neoformans* strains used in this study are listed in Supplementary Table S1. Strains were cultured on yeast extract–peptone–dextrose (YPD) medium [32]. 

### 2.2. Construction of Strains

To disrupt the *KU70* and *KU80* genes, information regarding the *KU70* (CNAG_04220) and *KU80* (CNAG_03637) genomic structures and sequences was obtained from FungiDB (https://fungidb.org/fungidb/, accessed on 5 August 2019). Primer pairs J1208/J1209 and J1210/J1211 were used for the amplification of the 5′- and 3′-flanking regions of the *KU70* gene, and primer pairs J1214/J1215 and J1216/J1217 were used for the amplification of the 5′- and 3′- flanking regions of the *KU80* gene, respectively, with the *C. neoformans* H99 genomic DNA as a template. The M13Fe and M13Re primers were used to amplify the Nar^r^ dominant selectable marker. The *KU70* and *KU80* gene disruption cassettes were generated by double-joint PCR (DJ-PCR), as previously described [33]. The gel-extracted gene disruption cassette was biolistically transformed into the *C. neoformans* strain. Next, stable transformants were selected on YPD medium containing nourseothricin (100 μg/mL) and screened by diagnostic PCR. To construct *ku70*Δ *rad51*Δ and *ku80*Δ *rad51*Δ double mutants, the *RAD51* gene disruption cassette with NEO resistance marker was generated by DJ-PCR. Each gel-extracted gene deletion cassette was biolistically inserted into the *ku70*Δ and *ku80*Δ mutants. Stable transformants were selected on YPD medium containing G418 (100 μg/mL) and initially screened by diagnostic PCR with primers B79 and SO. To demonstrate the correct genotype of each strain, Southern blot analysis was performed, as previously described (Figure S1) [34].

### 2.3. Construction of KU70, KU80, and RAD51 Complemented Strains

To demonstrate the phenotypes observed in the *ku70*Δ and *ku80*Δ mutants, the corresponding complemented strains were constructed as follows. The *KU70* gene was amplified separately by PCR using two fragments. A DNA fragment containing the promoter and 5′-region of the ORF was amplified by PCR using primers J1348 and J1349 with H99 genomic DNA as a template. Another fragment of the *KU70* gene was amplified by PCR using primers J1352 and J1353 with H99 genomic DNA as a template. Each PCR product was cloned into the plasmid pJET 1.2 (Thermo Fisher Scientific) to generate plasmids pJET-KU70L (KWE139) and pJET-KU70R (KWE131), respectively. Each clone was confirmed to have no errors in the sequence. The KpnI-digested pJET-KU70L insert was subcloned into pJET-KU70R to generate the plasmid pJET-KU70LR (KWE142). Next, the NotI-digested pJET-KU70LR insert was subcloned into pJET-HYG containing the Hyg^r^ marker to generate the plasmid pHYG-KU70 (KWE152). The pHYG-KU70 plasmid was linearized by NruI digestion and then biolistically transformed into *ku70*Δ mutants (KW1038). For *ku80*Δ+*KU80* strains, the two split fragments of the *KU80* gene containing its promoter, ORF, and terminator were separately amplified by PCR using the primer pairs J1342/J1343 and J1345/J1346, respectively. The PCR fragment containing the promoter and 5′-region of the exon of *KU80* amplified by PCR with primer pairs J1342/J1343 was cloned into the plasmid pJET 1.2, to generate the plasmid pJET-KU80L (KWE132). The PCR fragment containing the 3′-region of the exon of *KU80* and terminator amplified by PCR with primer pairs J1345/J1346 was cloned into the plasmid pJET 1.2, to generate the plasmid pJET-KU80R (KWE134). After sequencing to identify a clone with no error, the NdeI-digested pJET-KU80L insert was subcloned into pJET-KU80R to generate the plasmid pJET-KU80LR (KWE143). Next, the NotI-digested pJET-KU80LR insert was subcloned into pJET-HYG to generate the plasmid pHYG-KU80 (KWE148). pHYG-KU80 was linearized by BamHI digestion and then biolistically transformed into *ku80*Δ mutants (KW989). The integration of *KU70*-*HYG* and *KU80*-*HYG* alleles into the native locus of each gene was confirmed by diagnostic PCR. To construct a *RAD51* complemented strain, three PCR products (the promoter of *RAD51* [RAD51p], 5′-region of *RAD51* ORF with HA tagging sequence [RAD51L-HA], and the 3′-region and its terminator of *RAD51* [RAD51Rt]) were separately amplified by PCR with the primers listed in Table S2. RAD51p and RAD51L-HA were fused using an overlap PCR method with primer pairs J1271/J1274 from each PCR product as a template. Next, the overlap PCR product was cloned into pJET 2.1 to construct the plasmid pJET-RAD51L (KWE126). RAD51Rt was cloned into pJET 2.1 to construct the plasmid pJET-RAD51R (KWE130). The KpnI-digested pJET-RAD51R insert was subcloned into pJET-RAD51L to produce pJET-RAD51HA (KWE137). The NotI-cut pJET-RAD51HA was subcloned into the pJET-HYG to generate pHYG-RAD51HA (KWE135). The integration of pHYG-RAD51HA was delivered into the *rad51*Δ mutant (KW1213) using the biolistic transformation method. The integration of pHYG-RAD51HA was confirmed using diagnostic PCR. 

### 2.4. Spotting Assay

To investigate the roles of Ku70, Ku80, and Rad51 in the DNA damage response and oxidative stress, survival tests were performed using *ku70*Δ, *ku80*Δ, *ku70*Δ *rad51*Δ, and *ku80*Δ *rad51*Δ mutants with diverse stress inducers. Each strain was cultured in liquid YPD medium at 30 °C for 16 h, washed, and serially diluted (1 to 10^4^ dilutions). Next, 3 μL of cells was spotted onto solid YPD medium containing the indicated concentrations of stress inducers. The cells were further incubated at 30 °C for 4 days and photographed daily. 

### 2.5. Total RNA Isolation, cDNA Synthesis, and Real-Time Quantitative PCR (qRT-PCR)

To determine whether the expression levels of *KU70* and *KU80* genes were regulated by the Rad53–Bdr1 pathway after radiation exposure, total RNA was isolated from the wild type, *rad53*Δ, and *bdr1*Δ mutants as follows. Each strain was grown in 40 mL of liquid YPD medium for 16 h at 30 °C. Next, the grown cells were inoculated into 100 mL of fresh YPD medium and adjusted to OD_600_ = 0.2. Then, the cells were further incubated until OD_600_ reached approximately 0.6–0.7. Fifty milliliters of the cells was pelleted for the zero-time sample and the remaining cells were exposed to γ-radiation. After radiation exposure, 50 mL of the cells was further incubated at 30° C for 0.5 h. Total RNA was isolated using Trizol reagent (EasyBlue) as previously described [35] and further purified using RNeasy spin column (Qiagen) with RNase-free DNAse set to remove gDNA according to the manufacturer’s procedure. Next, cDNA was synthesized using the PrimeScript™ 1st strand cDNA Synthesis Kit (Takara Bio) using the purified RNA as a template. To investigate the relative expression levels of *KU70* and *KU80* genes under genotoxic stress, total RNA was isolated from cells treated with the indicated concentrations of MMS, bleomycin, and 4-NQO. Next, qRT-PCR analysis was performed with gene-specific primers listed in Table S2 using the CFX96 real-time PCR detection system (Bio-Rad). Relative expression of the target genes was determined using the 2^-∆∆Ct^ method with *ACT1* gene as an internal control [29,30,36], and statistical analyses were performed using one-way analysis of variance (ANOVA) with Bonferroni’s multiple-comparison test (GraphPad Software Inc., Sandiego, CA, USA).

### 2.6. Mutation Rate Assay

To measure the mutation frequency in the *rad51*Δ and *ku70*Δ/*ku80*Δ mutants, a fluctuation assay was performed. Each strain was cultured overnight in liquid YPD medium. Next, 10^5^ cells from the overnight culture were inoculated into fresh YPD medium and then cultured for 48 h at 30 °C. After growth for 48 h, 10^7^ cells were spread onto the solid YNB media containing 5-FOA (1 mg/mL) and uracil (0.05 mg/mL) [37]. The number of spontaneous colonies was counted, and the mutation rate was determined using statistical analysis.

### 2.7. Fluconazole Resistance Assay

To determine the frequency of antifungal drug resistance, each strain was cultured in liquid YPD medium for 16 h. Then, 10^5^ cells of each strain from the overnight culture were inoculated in three separate liquid YPD media and incubated for 48 h. Next, 10^6^ cells were spread on solid YPD medium containing 45 μg/mL fluconazole and further incubated at 30 °C for 5 days. The frequency of fluconazole resistance was determined as the number of colonies on the plate containing fluconazole divided by the total number of input cells (10^6^ cells). Statistical analyses were performed using one-way analysis of variance (ANOVA) with Bonferroni’s multiple-comparison test (GraphPad Software Inc.) [37].

### 2.8. Phenotypic Characterization of Microevolution of C. neoformans Strains

To demonstrate whether HR and NHEJ affect phenotypic changes during repeated passages, a protocol described in previous studies, with minor modifications, was followed [37,38]. Single colonies of the WT, *rad51*Δ, *ku70*Δ, *ku80*Δ, *rad51*Δ *ku70*Δ, and *rad51*Δ *ku80*Δ strains were re-streaked every 3 days for 3 months (approximately more than 1000 generations) on YPD plates at 30 °C. Three independent strains were generated for each tested strain. Every month, the three independent strains were sampled, and phenotypic changes were tested in response to diverse stresses, including DNA damage stress, oxidative stress, and antifungal drug treatment.

### 2.9. Identification of DNA Mutations

To identify DNA mutations in the *URA5* and *URA3* genes, genomic DNA from 5-FOA-resistant strains from the *rad51*Δ+*RAD51* strain and *rad51*Δ mutant was extracted using TENTS buffer [39]. The *URA5* and *URA3* genes were amplified using the primer pairs J1567/J1568 and J1623/J1624, respectively (Table S2). The PCR products were sequenced using J1149/J1150 and J1625/J1626, respectively.

## 3. Results

### 3.1. Expression of KU70 and KU80 Is Induced in Response to DNA Damage Stress in a Rad53–Bdr1 Pathway-Dependent Manner

**Figure 1:**
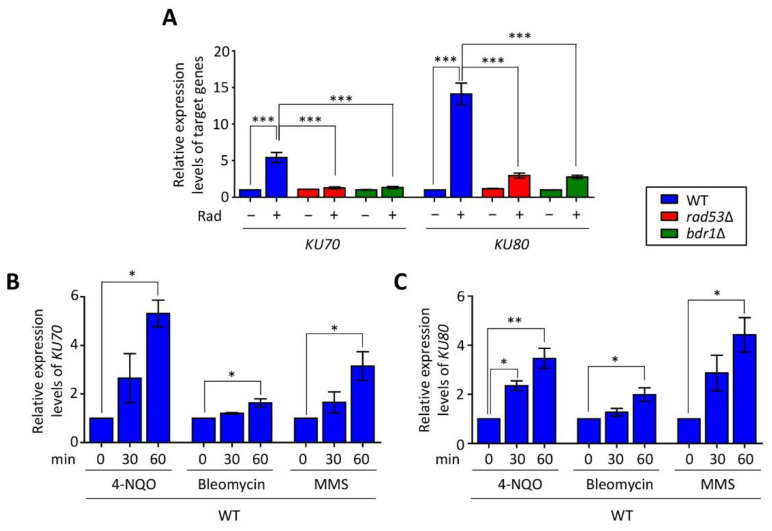
Expression levels of *KU70* and *KU80* in response to genotoxic stress. (**A**) Expressions of *KU70* and *KU80* were regulated by the Rad53–Bdr1 pathway. Real-time quantitative PCR (qRT-PCR) analysis using cDNA synthesized from total RNA isolated from wild-type (WT) strain, *rad53*Δ, and *bdr1*Δ mutants with or without gamma radiation exposure. (**B**,**C**) Expressions of *KU70* and *KU80* were gradually increased in the 4-NQO, bleomycin, and MMS treatments in the WT. Total RNA was isolated from the WT strain treated with the indicated concentration of 4-NQO (0.1 μg/mL), bleomycin (2 μg/mL), and MMS (0.02%) for 1 h, and cDNA was synthesized from these total RNA samples. Three independent biological experiments with duplicate technical replicates were performed. Statistical significance of difference was determined by one-way analysis of variance (ANOVA) with Bonferroni’s test. Error bars indicate standard errors of means (* *p* < 0.05, ** *p* < 0.01, and *** *p* < 0.001).

Our previous transcriptome analysis of the radiation-responsive pathway in *C. neoformans* showed that the expression levels of HR or NHEJ genes were highly upregulated [30]. In particular, the expression of *KU70* and *KU80* was regulated by Rad53 kinase, similar to *RAD51* expression. To demonstrate that the expression patterns of *KU70* and *KU80* genes are dependent on the Rad53–Bdr1 pathway, we measured the expression levels of these genes in WT, *rad53*Δ, and *bdr1*Δ mutants. Similar to transcriptome analysis, *KU70* and *KU80* genes in WT were highly increased after radiation exposure whereas those in the *rad53*Δ and *bdr1*Δ mutants did not increase (Figure 1A). Next, we wondered whether the expression of *KU70* and *KU80* genes is also increased in response to diverse types of DNA damage insults because NHEJ is known to mainly participate in the DSB repair process. We monitored the expression of *KU70* and *KU80* genes in response to a series of genotoxic DNA damage insults, such as 4-nitroquinoline 1-oxide (4-NQO, a DNA damage inducer through the production of reactive oxygen species), MMS (an inducer of DNA alkylation), and bleomycin (an inducer of DNA DSBs). Similar to the case in radiation exposure, expression levels of *KU70* and *KU80* genes gradually increased under 4-NQO, bleomycin, and MMS treatments, although their expression levels were distinct (Figure 1B,C). Taken together, *Cryptococcus KU70* and *KU80* genes were induced in response to DNA damage stress in a Rad53–Bdr1 pathway-dependent manner.

### 3.2. Ku70/Ku80 and Rad51 Cooperatively Regulate DNA Damage Response

**Figure 2:**
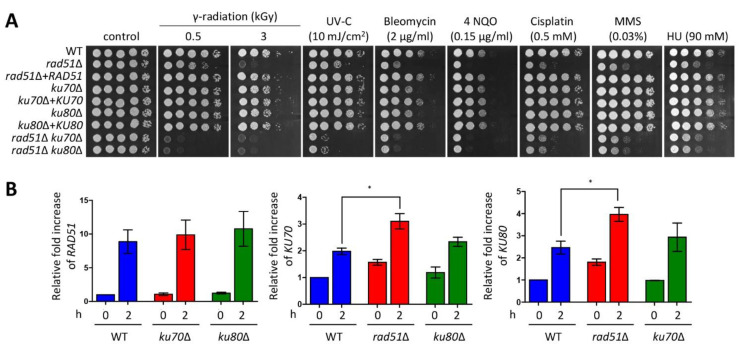
Rad51-mediated homologous recombination (HR) plays a major role in DNA repair. (**A**) Each *Cryptococcus neoformans* strain was cultured in liquid YPD medium for 16 h at 30 °C. The grown cells were serially diluted (1 to 10^4^) and then spotted on a YPD plate containing the indicated concentrations of DNA damage insults. For the gamma radiation and UV-C resistance test, the serially diluted cells spotted on the solid YPD plates were exposed to gamma radiation and UV-C. Strains were further incubated for 1 to 3 days at 30 °C and photographed daily. (**B**) The expression of *KU70* and *KU80* genes was higher in the *rad51*Δ mutant in response to bleomycin than that in the wild type (WT). Total RNA was isolated from the WT strain treated with the indicated concentration of bleomycin (2 μg/mL) for 2 h, and cDNA was synthesized from these total RNA samples. Three independent experiments were performed in duplicate. Statistical significance of difference was determined by one-way analysis of variance (ANOVA) with Bonferroni’s test. Error bars indicate standard errors of means (* *p* < 0.05).

A previous study reported that *C. neoformans ku70*Δ and *ku80*Δ mutants exhibited growth defects in the presence of phleomycin, but not other DNA damage insults, including MMS, UV, and HU [31]. In this study, to further elucidate the relationship between HR and NHEJ and characterize the role of genes belonging to each DNA repair pathway in *C. neoformans*, we constructed *ku70*Δ and *ku80*Δ mutants and *rad51*Δ *ku70*Δ and *rad51*Δ *ku80*Δ double mutants. Similar to the previous study, strains in which *KU70* and *KU80* genes were deleted were susceptible to bleomycin, whereas these strains were as resistant as the WT to other DNA damage agents (Figure 2A). The growth defect observed in the *rad51*Δ *ku70*Δ and *rad51*Δ *ku80*Δ mutants in response to bleomycin appeared to have an additive effect compared to the individual effects of gene deletion. However, *rad51*Δ *ku70*Δ and *rad51*Δ *ku80*Δ mutants were more sensitive to γ-radiation and UV-C exposure than each single mutant (Figure 2A). These data indicate that HR and NHEJ mediated by Rad51 and Ku70/Ku80, respectively, play redundant roles in DNA damage stresses induced by radiation and UV-C exposure. However, the *rad51*Δ mutant exhibited similar resistance in response to 4-NQO, cisplatin, MMS, and HU as *rad51*Δ *ku70*Δ and *rad51*Δ *ku80*Δ mutants (Figure 2A). Therefore, HR and NHEJ have redundant and distinct roles in a DNA damage type-dependent manner. 

HR is known to be the main pathway for DSB repair in the late S and G2 phases, whereas NHEJ contributes to other cell cycle phases. However, these two pathways appear to be complementary for DSB repair [20]. To elucidate the complementary relationship between HR and NHEJ, we monitored the expression levels of *RAD51*, *KU70*, and *KU80* genes in the WT, HR mutant (*rad51*Δ mutant), and NHEJ mutants (*ku70*Δ and *ku80*Δ mutants) under DNA damage stress. Expression levels of *RAD51* in the WT were similar to those in the *ku70*Δ and *ku80*Δ mutants in the presence or absence of bleomycin. Notably, *KU70* and *KU80* expression in the *rad51*Δ mutant was slightly increased compared to that in the WT under bleomycin-treated conditions (Figure 2B). Furthermore, we wondered whether this relationship between HR and NHEJ is also conserved under MMS treatment in which the agent did not inhibit growth additively in the *rad51*Δ *ku70*Δ and *rad51*Δ *ku80*Δ mutants compared to each single mutant. Similar to bleomycin treatment, induction levels of *RAD51* in both *ku70*Δ and *ku80*Δ mutants were similar to those in WT (Figure S2). However, the expression levels of *KU70* and *KU80* in the *rad51*Δ mutant were indistinguishable from those in the WT under MMS treatment. Taken together, HR is a more prominent pathway than NHEJ with regard to involvement in DNA damage repair. 

### 3.3. Ku70/Ku80 and Rad51 Are Involved in the Oxidative Stress Response

**Figure 3:**
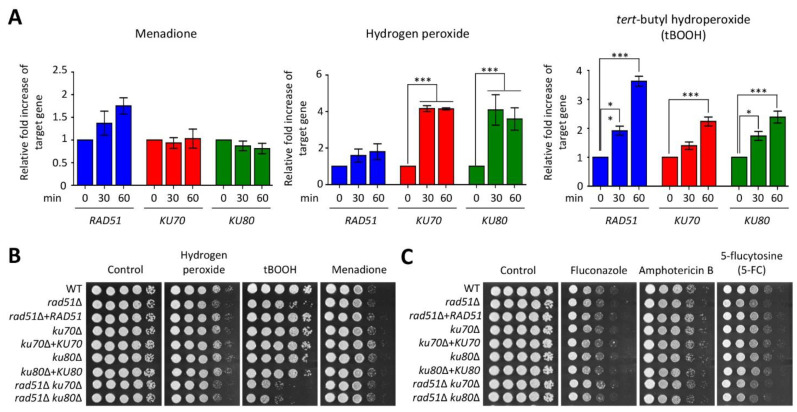
Functional roles of homologous recombination (HR) and non-homologous end joining (NHEJ) pathways in oxidative stress and antifungal drug resistance. (**A**) Expression levels of *KU70*, *KU80*, and *RAD51* genes in response to oxidative stress. Real-time quantitative PCR (qRT-PCR) analysis using cDNA synthesized from total RNA isolated from the wild-type (WT) strain treated with oxidative stress agents (hydrogen peroxide: 2.5 mM; tBOOH: 1 mM; menadione: 0.02 mM) for 1 h. Three independent biological experiments with duplicate technical replicates were performed. Statistical significance of difference was determined by ANOVA with Bonferroni’s test. Error bars indicate standard errors of means (* *p* < 0.05 and *** *p* < 0.001). (**B**,**C**) *C. neoformans* strains were cultured in liquid YPD medium 16 h at 30 °C. The grown cells were serially diluted (1 to 10^4^) and then spotted on a YPD plate containing the indicated concentration of oxidative stress agents (hydrogen peroxide: 2.5 mM; tBOOH: 0.9 mM; menadione: 0.02 mM) or antifungal drugs (fluconazole: 16 μg/mL; amphotericin B: 1 μg/mL; 5-flucytosine: 400 μg/mL). Strains were further incubated for 1 to 3 days at 30 °C and photographed daily.

In living organisms, intracellular ROS are generated during cellular processes, such as respiration, thereby leading to DNA damage stress [2]. To demonstrate whether HR- and NHEJ-mediated DNA repair systems are required for exogenous oxidative stress, we first measured the expression levels of these genes under a series of oxidative stress agents. Interestingly, in contrast to the response to DNA-damage-inducing agents, the expression of *RAD51*, *KU70*, and *KU80* was dependent on oxidative stress inducers. In the case of menadione, which is a superoxide generator, the expression levels of *RAD51*, *KU70*, and *KU80* were indistinguishable from those in the presence or absence of menadione (Figure 3A, left). In hydrogen peroxide treatment, *RAD51* expression was not significantly increased in a time-dependent manner, whereas the expression of both *KU70* and *KU80* was highly upregulated (Figure 3A, middle). Notably, the expression levels of *RAD51*, *KU70*, and *KU80* genes were gradually induced in a time-dependent manner under *tert*-butyl hydroperoxide (tBOOH) treatment (Figure 3A, right).

Given that *RAD51*, *KU70*, and *KU80* expression levels were altered in response to oxidative stresses, we performed survival assays using these mutants to elucidate the role of these genes in oxidative stress. Consistent with the expression pattern, the levels of resistance to menadione demonstrated by each HR and NHEJ single mutant and both double mutants were similar to those demonstrated by the WT (Figure 3B). However, the *rad51*Δ mutant, but not the *ku70*Δ and *ku80*Δ mutants, was susceptible to hydrogen peroxide, although its expression was not significantly increased. Deletion of *KU70* or *KU80* in the *rad51*Δ mutant did not render cells more susceptible to hydrogen peroxide (Figure 3B). This indicates that HR mediated by Rad51 contributes to oxidative stress resistance caused by hydrogen peroxide (Figure 3B). Similar to hydrogen peroxide, perturbation of *RAD51*, but not *KU70* and *KU80*, resulted in growth retardation following treatment with tBOOH. Notably, both *rad51*Δ *ku70*Δ and *rad51*Δ *ku80*Δ double mutants were more sensitive to tBOOH than each single mutant, suggesting that HR and NHEJ play redundant roles in the oxidative stress response induced by tBOOH (Figure 3B). A recent study revealed that fluconazole treatment leads to changes in the expression levels of DNA repair genes, such as *RAD54*, encoding a DNA-dependent ATPase, in *C. neoformans* [40]. Furthermore, our previous study revealed that perturbations of the DNA repair pathway mediated by Rad53 and Chk1 kinases render cells susceptible to antifungal drugs, including 5-FC and amphotericin B [30]. These results prompted us to perform an antifungal drug resistance assay using HR and NHEJ mutants. Unexpectedly, deletion of HR and NHEJ did not affect fluconazole and amphotericin B resistance (Figure 3C). We found that the *rad51*Δ mutant, but not the *ku70*Δ and *ku80*Δ mutants, was slightly sensitive to 5-FC. However, *rad51*Δ *ku70*Δ and *rad51*Δ *ku80*Δ double mutants showed similar levels of resistance to 5-flucytosine as those of the *rad51*Δ mutant, indicating that Ku70/Ku80-mediated NHEJ is not responsible for 5-FC resistance (Figure 3C).

### 3.4. Functional Role of Accumulation of DNA Mutations and Microevolution Mediated by HR and NHEJ

**Figure 4:**
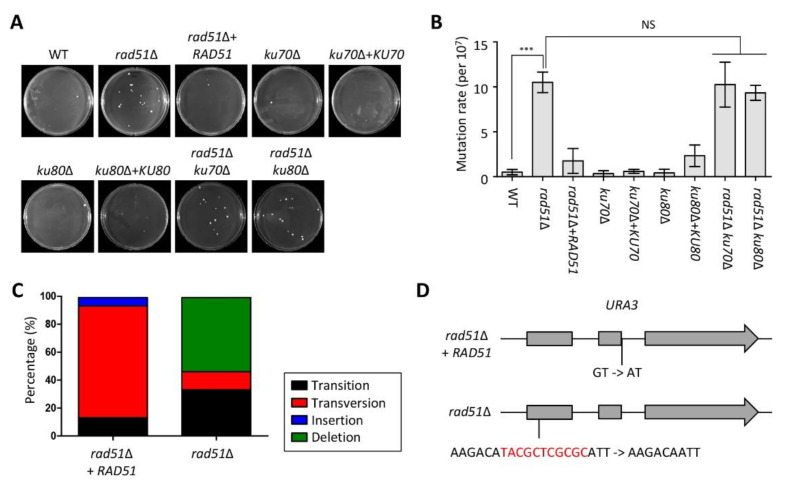
Deletion of *RAD51* increases the frequency of mutations. (**A**) Spontaneous 5-FOA-resistant colonies in the wild-type (WT) strain, *rad51*Δ, *rad51*Δ+*RAD51*, *ku70*Δ, *ku70*Δ+*KU70*, *ku80*Δ, *ku80*Δ+*KU80*, *rad51*Δ *ku70*Δ, and *rad51*Δ *ku80*Δ strains. (**B**) Quantification of spontaneous 5-FOA-resistant rates in the homologous recombination HR and NHEJ mutants. Three independent experiments with five samples were performed. Statistical significance of difference was determined by one-way analysis of variance (ANOVA) with Bonferroni’s test. Error bars indicate standard errors of means (*** *p* < 0.001). (**C**) Mutation profiling of 5-FOA-resistant strains isolated from *rad51*Δ+*RAD51* and *rad51*Δ mutants. (**D**) Schematic representation of *URA3* mutation in 5-FOA-resistant strains isolated from *rad51*Δ+*RAD51* and *rad51*Δ mutants. Nucleotides marked with red were deleted in the *URA3* gene in 5-FOA-resistant strain isolated from the *rad51*Δ mutant.

Several studies have reported that perturbations of DNA repair systems, such as mismatch repair, result in the accumulation of DNA mutations at high frequencies [37,38,41]. To demonstrate whether HR and NHEJ regulate the accumulation of DNA mutations similar to the mismatch repair system, we measured mutation rates using fluctuation analysis. The *rad51*Δ mutant exhibited more 5-FOA-resistant colonies compared to the WT and its complemented strain, indicating that Rad51 is involved in the accumulation of spontaneous mutations. However, the number of 5-FOA-resistant colonies in the *ku70*Δ and *ku80*Δ mutants was similar to that of the WT. Furthermore, the mutation rate in the *rad51*Δ *ku70*Δ and *rad51*Δ *ku80*Δ mutants was indistinguishable from that in the *rad51*Δ mutant (Figure 4A,B).

Next, most of the causes of resistance to 5-FOA are mutations in the *URA3* and *URA5* genes; in particular, the mutation pattern of DNA is dependent on the DNA repair system [37,42]. To determine the mutation patterns of both *URA3* and *URA5* genes in the *rad51*Δ mutant, we sequenced both genes using genomic DNA from the 15 5-FOA-resistant strains isolated from *rad51*Δ+*RAD51* and *rad51*Δ mutants. Many portions of *URA5* and *URA3* mutations in *rad51*Δ+*RAD51*-originated strains were point mutations derived from transversion (13%) and transition (80%), whereas those in *URA5* and *URA3* in *rad51*Δ mutant-originated strains were nucleotide deletions (53%) (Figure 4C). Interestingly, all transition mutations of *URA3* in 5-FOA-resistant strains from *rad51*Δ+*RAD51* occurred in the junction region between the second exon and intron. Likewise, 12 nucleotides were deleted in the same region of the first exon region from the 5-FOA-resistant strain derived from the *rad51*Δ mutant (Figure 4D). However, *URA5* mutations in the *rad51*Δ+*RAD51* and *rad51*Δ mutants occurred in diverse positions (Table S3).

Next, to determine whether HR and NHEJ are involved in the acquisition of drug-resistance-like fluctuations, we calculated the colony frequency of fluconazole-resistant strains. In contrast to the results of the fluctuation analysis, deletions of HR and NHEJ genes did not change the frequency of fluconazole resistance (Figure S3). These data indicate that HR and NHEJ are insufficient for increasing the frequency of fluconazole resistance.

**Figure 5:**
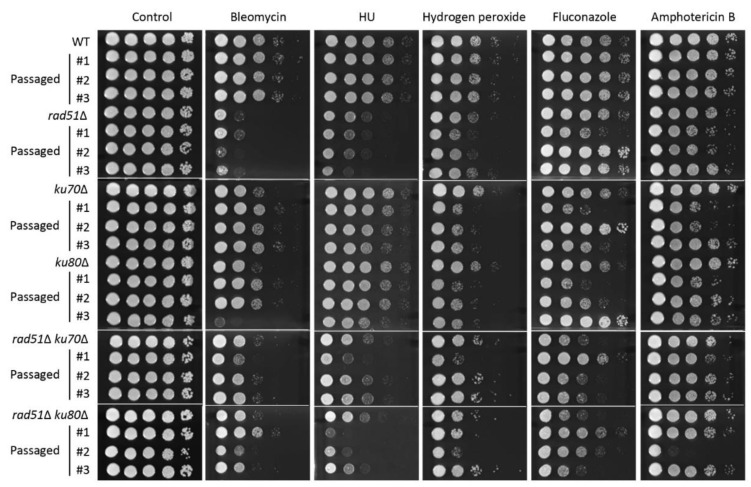
Lack of Rad51-mediated homologous recombination (HR) and KU70/KU80 results in rapid microevolution. The phenotypic changes of original wild-type (WT) strain, *rad51*Δ, *ku70*Δ, *ku80*Δ, *rad51*Δ *ku70*Δ, and *rad51*Δ *ku80*Δ mutants compared to those of its corresponding strains after three independent passages (about 600 generations) in response to diverse stress responses. Each strain was cultured in liquid YPD media at 30 °C. The cultured cells were serially diluted (1 to 10^4^) and spotted on the YPD plate containing stress agents. Cells were further incubated at 30 °C and photographed daily for 3 days. The two images split by a horizontal white line in each spot assay were obtained from the same plate.

Previous studies have reported that disruption of the mismatch repair pathway and the lack of DNA polymerase, *POL3*, lead to rapid microevolution [37,38]. Furthermore, the result that perturbation of *RAD51* increased mutation rate in the fluctuation assay led us to investigate whether HR and NHEJ are involved in microevolution. To determine the roles of HR and NHEJ components in microevolution, we compared phenotypic changes in the original WT, *rad51*Δ, *ku70*Δ, *ku80*Δ, *rad51*Δ *ku70*Δ, and *rad51*Δ *ku80*Δ mutants to those of the corresponding three independent passage strains depending on the number of generations (around 300, 600, and 1000 generations) in response to diverse stress responses, including oxidative stress, DNA damage stress, and antifungal drug resistance. In approximately 300 generations, passaged strains from *rad51*Δ, *rad51*Δ *ku70*Δ, and *rad51*Δ *ku80*Δ mutants exhibited different degrees of stress resistance in response to bleomycin and HU compared to the original strain (Figure S4A). However, the passaged strains (about 300 generations) from WT, *ku70*Δ, and *ku80*Δ mutants showed similar phenotypes as those of the original strain (Figure S4A). In approximately 600 generations, passaged strains from *ku70*Δ and *ku80*Δ mutants also showed phenotypic changes under bleomycin, HU, hydrogen peroxide, fluconazole, and amphotericin B treatment (Figure 5). Notably, passaged strains (about 600 generations) from *rad51*Δ *ku70*Δ and *rad51*Δ *ku80*Δ mutants did not show additional phenotypic variations those from the *rad51*Δ, *ku70*Δ, and *ku80*Δ mutants. However, passaged strains from the WT strain still showed WT levels of stress resistance until 600 generations, and phenotypic changes between WT and its passaged strains were observed after 1000 generations (Figure S4B). Collectively, HR and NHEJ control genome stability in *C. neoformans*.

## 4. Discussion

Although HR and NHEJ play overlapping roles in DSB repair, the contribution of each pathway to DNA damage stress is evolutionarily divergent. In mammals, NHEJ plays a dominant role in DSB repair, whereas HR plays a more critical role in DSB repair than NHEJ in fungi [43,44,45]. Supporting this notion, the *C. neoformans rad51*Δ mutant was more susceptible to diverse types of genotoxic stress than the *ku70*Δ and *ku80*Δ mutants. Although NHEJ plays a minor role in the DNA damage stress in fungi, the phenotypes conferred by NHEJ mutations vary depending on the species. In *S. cerevisiae*, deletion of *KU70* results in additional sensitivity in response to γ-radiation and MMS in *rad52*Δ mutant [44]. Unlike *S. cerevisiae*, the resistance of the *ku70*Δ mutant in response to MMS and UV-C is indistinguishable from that of the *rad52*Δ *ku70*Δ double mutant in *C. albicans* [43]. Notably, the strain deleted with only *KU80* shows growth defects in response to HU, 4-NQO, MMS, and camptothecin in *Aspergillus fumigatus* [46]. In *C. neoformans*, each *ku70*Δ and *ku80*Δ mutant was susceptible to bleomycin, whereas both *rad51*Δ *ku70*Δ and *rad51*Δ *ku80*Δ mutants exhibited different levels of growth defects than their single-mutant counterparts, depending on the DNA damage stress inducers.

Although it is well known that HR and NHEJ participate separately in DSB repair in a cell-cycle-dependent manner, the activation of HR and NHEJ is connected. Several studies have reported that the frequency of HR-mediated recombination is increased in the absence of NHEJ in fungal species [31,47,48]. Similarly, the frequency of DSB repair by HR was elevated in NHEJ mutants in mammals [49]. These data indicate that the HR pathway compensates for recombination in the absence of the NHEJ pathway. In agreement with the previous results, our study also showed that the expression levels of *KU70* and *KU80* genes were slightly higher in the *rad51*Δ mutant than in the WT in response to bleomycin. This transcriptional compensation occurs when their synthetic lethal or paralog genes are lost [50]. In *S. cerevisiae*, disruption of *FKS1*, encoding glucan synthase for the synthesis of beta-1,3-glucan in the cell wall, induces an increase in the expression of genes encoding other glycosylphosphatidylinositol (GPI)-dependent cell wall proteins, such as *YPS3*, *CRH1*, *PST1*, and *CWP1* [51]. Therefore, the HR and NHEJ pathways also serve as connective genetic backup circuits for the DNA damage response.

In humans, defects in DNA damage repair systems increase the rates of mutations or genome rearrangements, thereby inducing diseases, such as cancer. The perturbation of the DNA repair system accelerates the emergence of drug-resistant strains in fungal pathogens, such as *C. glabrata* and *C. neoformans* [24,37,38]. For this reason, the relationship between the DNA repair system and the emergence of antifungal drug resistance has been highlighted. In this study, we revealed that the deletion of HR and NHEJ did not affect the frequency of antifungal drug resistance. Similarly, in *C. albicans*, strains deleted with NHEJ genes, such as *KU80*, or HR genes, *RAD52*, showed WT levels of fluconazole-resistant colonies, whereas strains lacking MMR genes, such as *MSH2* and *PMS1*, displayed a higher number of fluconazole-resistant colonies [52]. Furthermore, *C. albicans* strains with deletions in BER or NER genes did not exhibit changes in the appearance of fluconazole-resistant colonies [53]. These data suggest that DSB repair mediated by HR and NHEJ and excision repair mediated by BER and NER do not contribute to genomic changes associated with the acquisition of drug resistance. In addition to its role in altering the frequency of drug resistance mediated by HR and NHEJ, we further revealed that HR, but not NHEJ, was required for 5-FC resistance. Although the fungistatic mechanism of 5-FC is relevant to DNA damage, strains with a deleted HR system in *C. albicans* and *Candida auris* showed WT levels of 5-FC resistance in contrast to *C. neoformans* [54,55]. These data indicate that DNA repair systems involved in antifungal drug resistance are species dependent. 

Similar to previous studies [37,38], our study also reported that impairment of the DNA repair system mediated by HR or NHEJ leads to an increased mutation rate and rapid microevolution. However, the patterns of mutation rate and microevolution observed in the HR and NHEJ mutants showed similarities and discrepancies with those of MMR and *pol3^D270G^* strains. First, the *RAD51*-deleted strain generated more 5-FOA-resistant strains than the WT, whereas *KU70*- or *KU80*-deficient strains exhibited mutation rates similar to the WT. Notably, the number of 5-FOA-resistant colonies in the background of strains deleted with both *RAD51* and *KU70* appeared to be similar to that of the *rad51*Δ mutant. This result is in stark contrast to the phenotypes of *rad51*Δ *ku70*Δ and *rad51*Δ *ku80*Δ mutants in response to DNA damage stress. This phenomenon could not be explained by the reduced viability of *rad51*Δ *ku70*Δ and *rad51*Δ *ku80*Δ mutants to toxic materials because these strains were as resistant to 5-FOA as the *rad51*Δ mutant. Second, the mutation profile in the HR mutant was different from that in the MMR mutants. Strains with deleted *MLH1*, *MSH2*, or *PMS1* and the strain mutated with *pol3^D270G^* have more transition mutations than insertion or deletion (indel) mutations [37,38]. In the case of *RAD51* deletion, indel mutations were more obvious than transition and transversion mutations. 

In this study, we demonstrated that the Rad51-mediated HR and Ku70/Ku80-mediated NHEJ pathways cooperatively repair DNA damage. Among these pathways, HR plays a major role in DNA damage repair, rather than NHEJ, in other fungal species. Furthermore, we found that HR and NHEJ are involved in the oxidative stress response. Notably, we confirmed that the perturbation of HR or NHEJ resulted in accelerated microevolution. Taken together, this study provides insights into the potential roles of the HR and NHEJ pathways in DNA damage response and microevolution in human fungal pathogens.

## Data Availability

The strains presented in this study are available on request from the corresponding author.

## Supplementary Materials

The following are available online at https://www.mdpi.com/article/10.3390/jof7070566/s1, Figure S1: Construction of *ku70*Δ, *ku80*Δ, *rad51*Δ *ku70*Δ and *rad51*Δ *ku80*Δ double mutants; Figure S2: Expressions of *KU70* and *KU80* were not changed in the *rad51*Δ mutant in response to MMS compared to those of the wild type (WT); Figure S3: HR and NHEJ were not involved in the frequency of fluconazole resistance; Figure S4: Lack of Rad51-mediated homologous recombination (HR) and *KU70*/*KU80*-mediated non-homologous end joining (NHEJ) resulted in rapid microevolution. Table S1: Strains used in this study; Table S2: Primers used in this study; Table S3: Mutation profiles in the 5-FOA-resistant strains.
