# Supplementary material for: Functional Roles of Homologous Recombination and Non-Homologous End Joining in DNA Damage Response and Microevolution in Cryptococcus neoformans
Source: J Fungi (Basel). 2021 Jul 16;7(7):566. doi: 10.3390/jof7070566 (PMC8307084; doi:10.3390/jof7070566)
Supplement: Supplementary file 1 [file jof-07-00566-s001.zip › Fig_S1_Jung et al.pptx]

## Slide 1
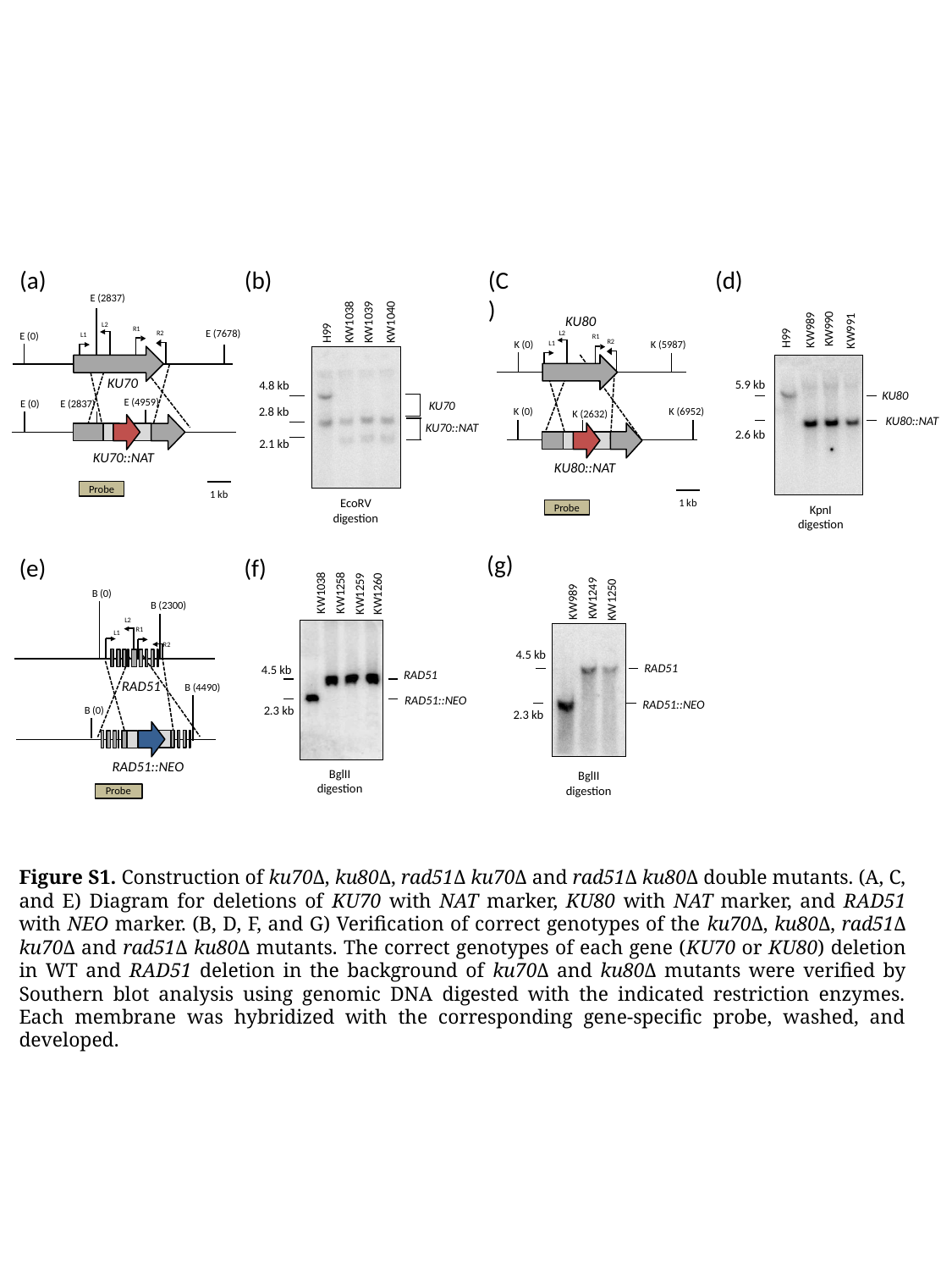

(a)
(b)
(C)
(d)
E (2837)
H99
KW1038
KW1039
KW1040
KW990
KW989
H99
KW991
KU80
L2
R1
E (7678)
L2
R2
E (0)
L1
R1
R2
K (5987)
K (0)
L1
KU70
5.9 kb
4.8 kb
KU80
E (4959)
E (0)
E (2837)
KU70
2.8 kb
K (6952)
K (0)
K (2632)
KU80::NAT
KU70::NAT
2.6 kb
2.1 kb
KU70::NAT
KU80::NAT
Probe
1 kb
EcoRV
digestion
1 kb
Probe
KpnI
digestion
(g)
(e)
(f)
KW1258
KW1038
KW1259
KW1260
KW1249
KW989
KW1250
B (0)
B (2300)
L2
R1
L1
R2
4.5 kb
RAD51
4.5 kb
RAD51
RAD51
B (4490)
RAD51::NEO
RAD51::NEO
2.3 kb
B (0)
2.3 kb
RAD51::NEO
BglII
digestion
BglII
digestion
Probe
Figure S1. Construction of ku70Δ, ku80Δ, rad51Δ ku70Δ and rad51Δ ku80Δ double mutants. (A, C, and E) Diagram for deletions of KU70 with NAT marker, KU80 with NAT marker, and RAD51 with NEO marker. (B, D, F, and G) Verification of correct genotypes of the ku70Δ, ku80Δ, rad51Δ ku70Δ and rad51Δ ku80Δ mutants. The correct genotypes of each gene (KU70 or KU80) deletion in WT and RAD51 deletion in the background of ku70Δ and ku80Δ mutants were verified by Southern blot analysis using genomic DNA digested with the indicated restriction enzymes. Each membrane was hybridized with the corresponding gene-specific probe, washed, and developed.
