# Supplementary material for: Functional Roles of Homologous Recombination and Non-Homologous End Joining in DNA Damage Response and Microevolution in Cryptococcus neoformans
Source: J Fungi (Basel). 2021 Jul 16;7(7):566. doi: 10.3390/jof7070566 (PMC8307084; doi:10.3390/jof7070566)
Supplement: Supplementary file 1 [file jof-07-00566-s001.zip › Fig_S2_Jung et al.pptx]

## Slide 1
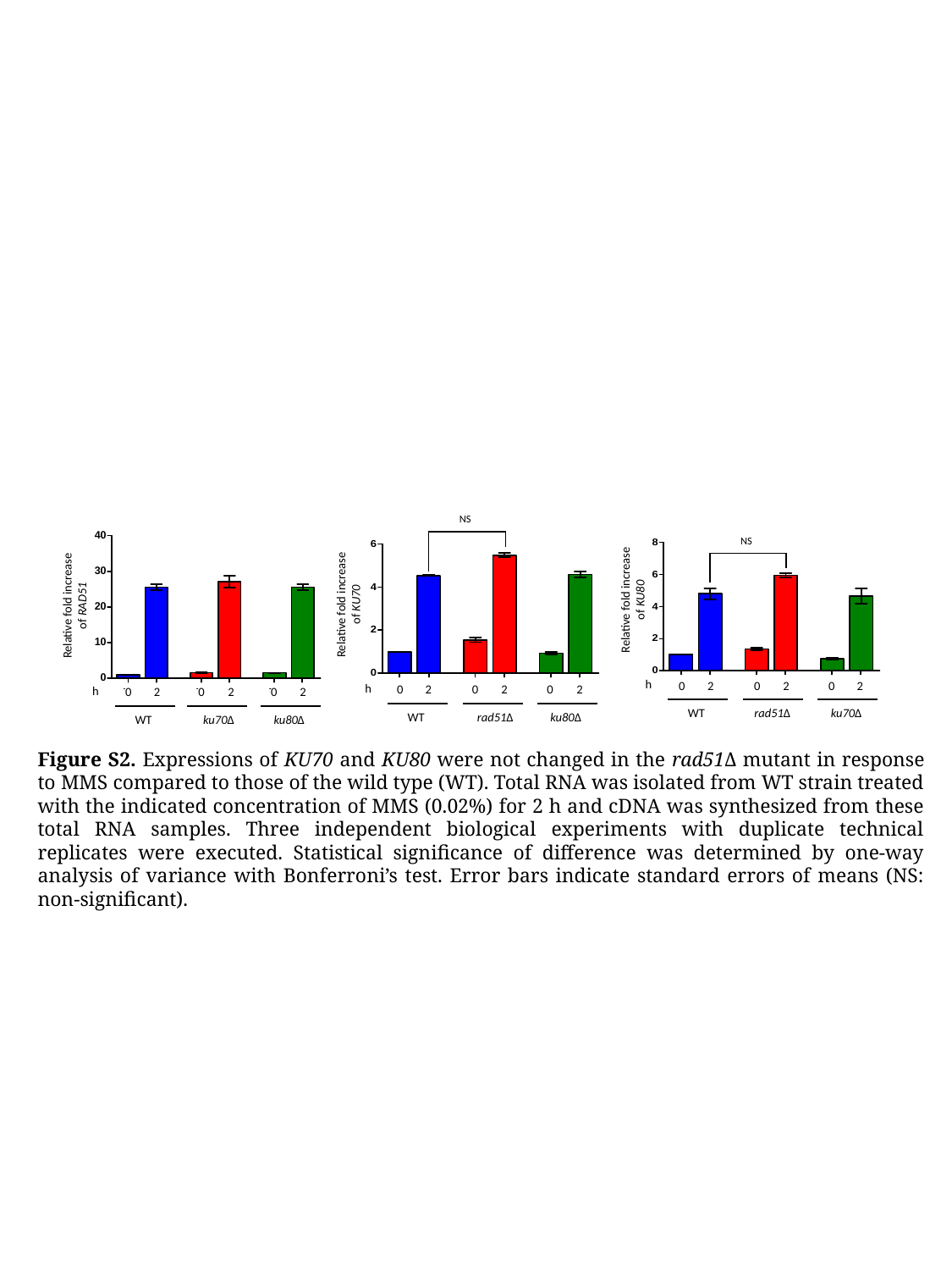

NS
NS
Relative fold increase
of KU80
Relative fold increase
of KU70
Relative fold increase
of RAD51
h
0
2
0
2
0
2
h
0
2
0
2
0
2
h
0
2
0
2
0
2
WT
rad51∆
ku70∆
WT
rad51∆
ku80∆
WT
ku70∆
ku80∆
Figure S2. Expressions of KU70 and KU80 were not changed in the rad51Δ mutant in response to MMS compared to those of the wild type (WT). Total RNA was isolated from WT strain treated with the indicated concentration of MMS (0.02%) for 2 h and cDNA was synthesized from these total RNA samples. Three independent biological experiments with duplicate technical replicates were executed. Statistical significance of difference was determined by one-way analysis of variance with Bonferroni’s test. Error bars indicate standard errors of means (NS: non-significant).
