# Supplementary material for: Functional Roles of Homologous Recombination and Non-Homologous End Joining in DNA Damage Response and Microevolution in Cryptococcus neoformans
Source: J Fungi (Basel). 2021 Jul 16;7(7):566. doi: 10.3390/jof7070566 (PMC8307084; doi:10.3390/jof7070566)
Supplement: Supplementary file 1 [file jof-07-00566-s001.zip › Fig_S3_Jung et al.pptx]

## Slide 1
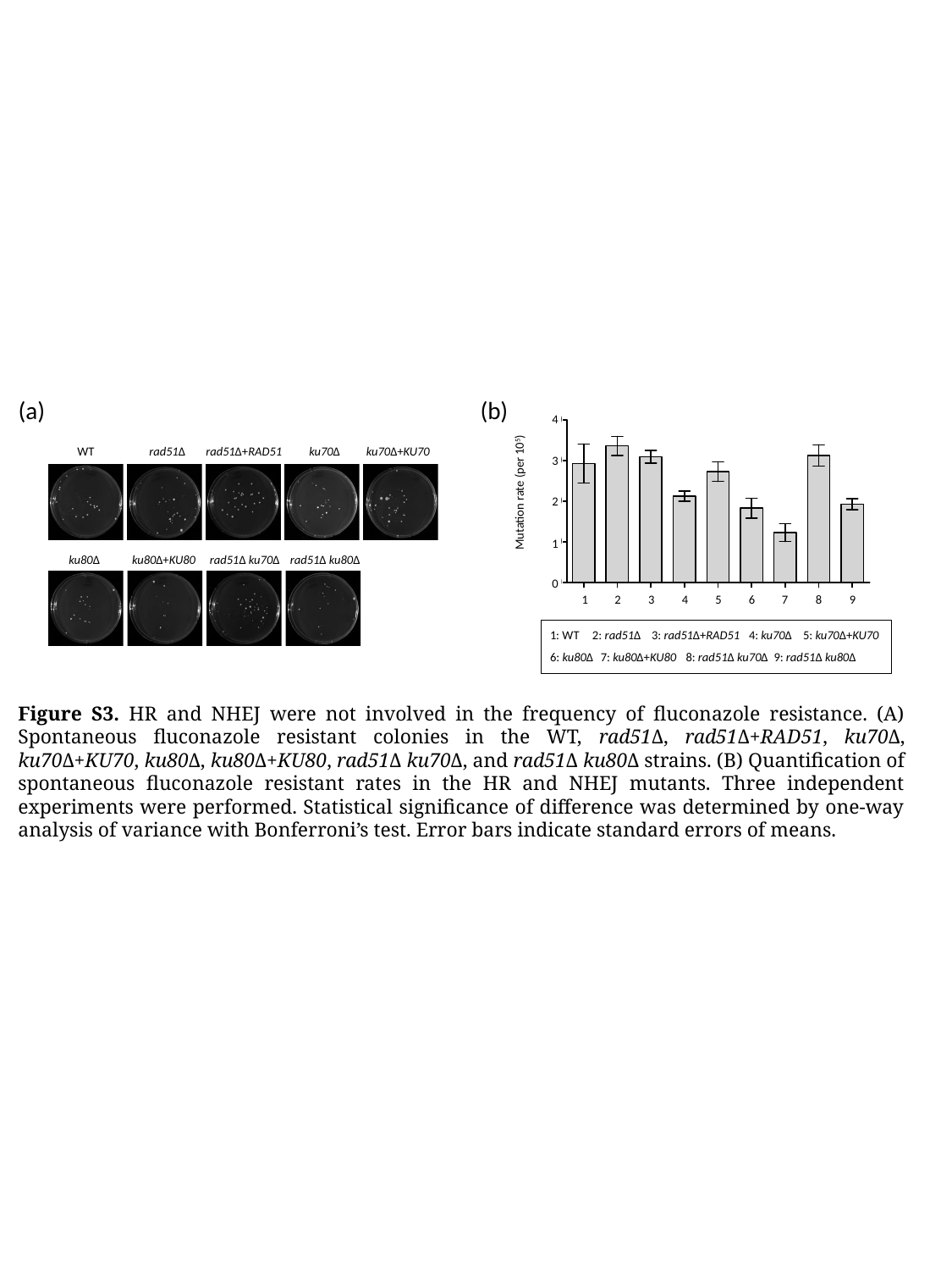

(b)
(a)
4
WT
rad51∆
rad51∆+RAD51
ku70∆
ku70∆+KU70
3
Mutation rate (per 105)
2
1
ku80∆
ku80∆+KU80
rad51∆ ku70∆
rad51∆ ku80∆
0
1
2
3
4
5
6
7
8
9
1: WT
2: rad51∆
3: rad51∆+RAD51
4: ku70∆
5: ku70∆+KU70
6: ku80∆
7: ku80∆+KU80
8: rad51∆ ku70∆
9: rad51∆ ku80∆
Figure S3. HR and NHEJ were not involved in the frequency of fluconazole resistance. (A) Spontaneous fluconazole resistant colonies in the WT, rad51Δ, rad51Δ+RAD51, ku70Δ, ku70Δ+KU70, ku80Δ, ku80Δ+KU80, rad51Δ ku70Δ, and rad51Δ ku80Δ strains. (B) Quantification of spontaneous fluconazole resistant rates in the HR and NHEJ mutants. Three independent experiments were performed. Statistical significance of difference was determined by one-way analysis of variance with Bonferroni’s test. Error bars indicate standard errors of means.
