# Supplementary material for: Functional Roles of Homologous Recombination and Non-Homologous End Joining in DNA Damage Response and Microevolution in Cryptococcus neoformans
Source: J Fungi (Basel). 2021 Jul 16;7(7):566. doi: 10.3390/jof7070566 (PMC8307084; doi:10.3390/jof7070566)
Supplement: Supplementary file 1 [file jof-07-00566-s001.zip › Fig_S4_Jung et al.pptx]

## Slide 1
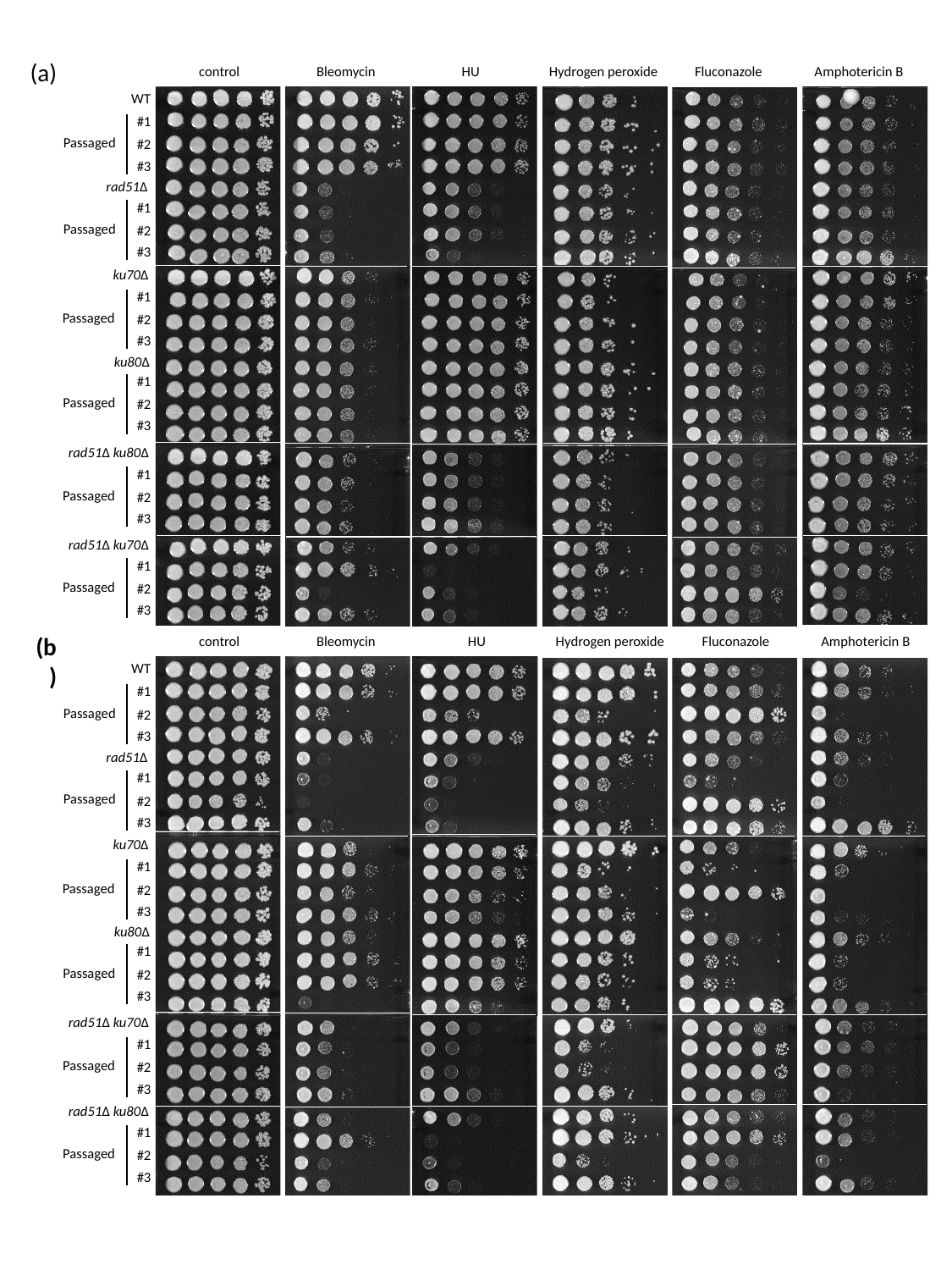

(a)
control
Bleomycin
HU
Hydrogen peroxide
Fluconazole
Amphotericin B
WT
#1
Passaged
#2
#3
rad51∆
#1
Passaged
#2
#3
ku70∆
#1
Passaged
#2
#3
ku80∆
#1
Passaged
#2
#3
rad51∆ ku80∆
#1
Passaged
#2
#3
rad51∆ ku70∆
#1
Passaged
#2
#3
(b)
control
Bleomycin
HU
Hydrogen peroxide
Fluconazole
Amphotericin B
WT
#1
Passaged
#2
#3
rad51∆
#1
Passaged
#2
#3
ku70∆
#1
Passaged
#2
#3
ku80∆
#1
Passaged
#2
#3
rad51∆ ku70∆
#1
Passaged
#2
#3
rad51∆ ku80∆
#1
Passaged
#2
#3

## Slide 2
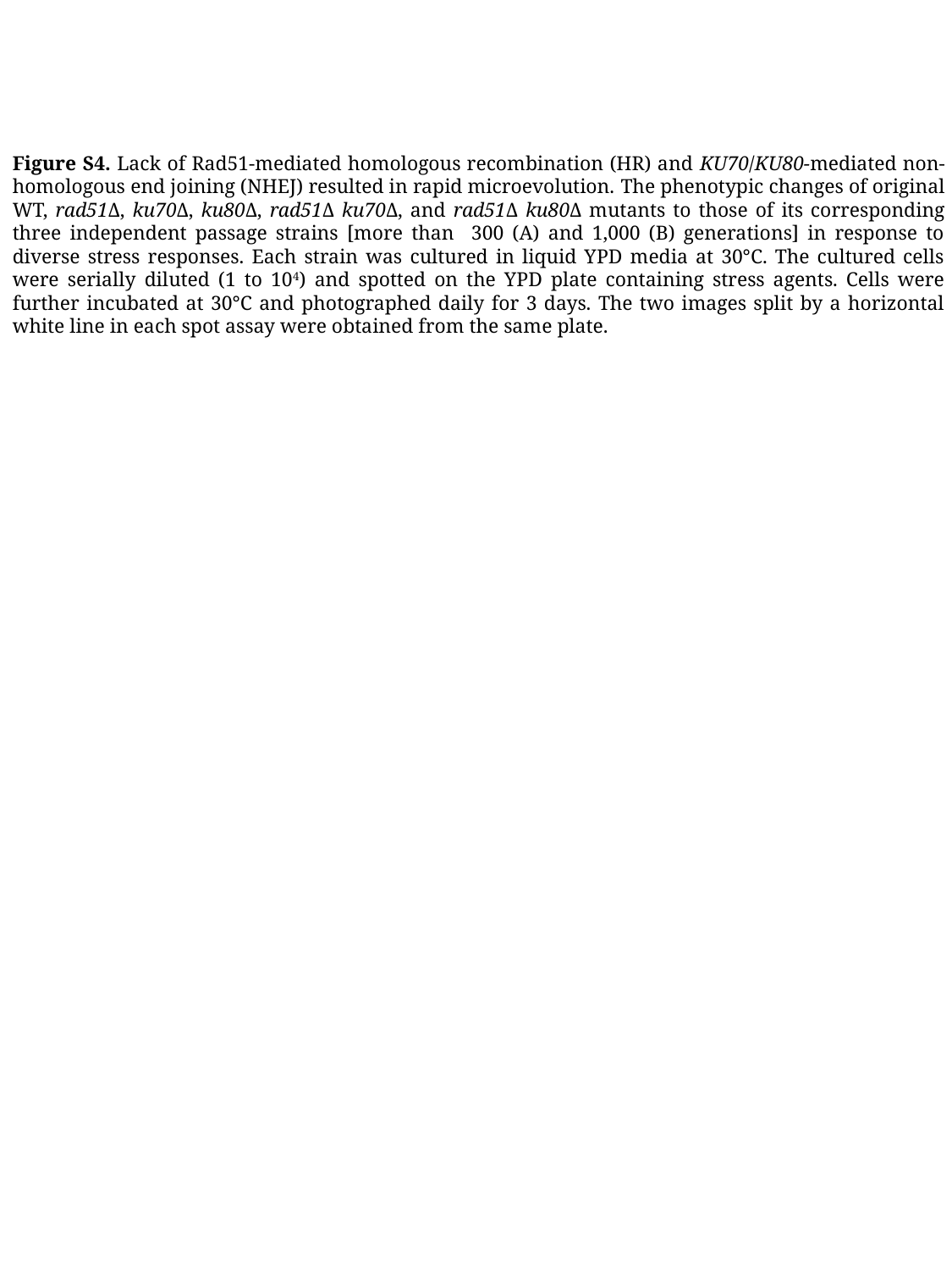

Figure S4. Lack of Rad51-mediated homologous recombination (HR) and KU70/KU80-mediated non-homologous end joining (NHEJ) resulted in rapid microevolution. The phenotypic changes of original WT, rad51Δ, ku70Δ, ku80Δ, rad51Δ ku70Δ, and rad51Δ ku80Δ mutants to those of its corresponding three independent passage strains [more than 300 (A) and 1,000 (B) generations] in response to diverse stress responses. Each strain was cultured in liquid YPD media at 30°C. The cultured cells were serially diluted (1 to 104) and spotted on the YPD plate containing stress agents. Cells were further incubated at 30°C and photographed daily for 3 days. The two images split by a horizontal white line in each spot assay were obtained from the same plate.
