# Supplementary material for: Functional Roles of Homologous Recombination and Non-Homologous End Joining in DNA Damage Response and Microevolution in Cryptococcus neoformans
Source: J Fungi (Basel). 2021 Jul 16;7(7):566. doi: 10.3390/jof7070566 (PMC8307084; doi:10.3390/jof7070566)
Supplement: Supplementary file 1 [file jof-07-00566-s001.zip › Table_S1_Jung et al.pdf]

Table S1. Strains used in this study

Strain	Genotype	Parent	Reference
H99	*MATα*		[1]
KW137	*MATα CNAG_02589* (*BDR1*)*::NAT #230*	H99	[2]
YSB3785	*MATα CNAG_05216* (*RAD53*)*::NAT#184*	H99	[3]
KW1213	*MATα CNAG_00720::NAT#43* (*RAD51*)	H99	[2]
KW1239	*MATα CNAG_00720* (*RAD51*)*::NAT# pHYG-RAD51-N-HA*	KW1213	This study
KW1038	*MATα CNAG_04220::NAT#43 (KU70)*	H99	This study
KW1039	*MATα CNAG_04220::NAT#43 (KU70)*	H99	This study
KW1040	*MATα CNAG_04220::NAT#43 (KU70)*	H99	This study
KW1276	*MATα CNAG_04220::NAT#43 (KU70) pHYG-KU70*	KW1038	This study
KW989	*MATα CNAG_03637::NAT#234* (*KU80*)	H99	This study
KW990	*MATα CNAG_03637::NAT#234* (*KU80*)	H99	This study
KW991	*MATα CNAG_03637::NAT#234* (*KU80*)	H99	This study
KW1272	*MATα CNAG_03637::NAT#234* (*KU80*) *pHYG-KU80*	KW989	This study
KW1258	*MATα CNAG_04220::NAT#43* (*KU70*) *CNAG_00720::NEO (RAD51)*	KW1038	This study
KW1259	*MATα CNAG_04220::NAT#43* (*KU70*) *CNAG_00720::NEO (RAD51)*	KW1038	This study
KW1260	*MATα CNAG_04220::NAT#43* (*KU70*) *CNAG_00720::NEO (RAD51)*	KW1038	This study
KW1249	*MATα CNAG_03637::NAT#234* (*KU80*) *CNAG_00720::NEO (RAD51)*	KW989	This study
KW1250	*MATα CNAG_03637::NAT#234* (*KU80*) *CNAG_00720::NEO (RAD51)*	KW989	This study

Each *NAT-STM#* indicates the Nat^r marker with a unique signature tag.

References

1. Perfect, J.R.; Ketabchi, N.; Cox, G.M.; Ingram, C.W.; Beiser, C.L. Karyotyping of *Cryptococcus neoformans* as an epidemiological tool. *J. Clin. Microbiol.* **1993**, *31*, 3305-3309.
2. Jung, K.W.; Yang, D.H.; Kim, M.K.; Seo, H.S.; Lim, S.; Bahn, Y.S. Unraveling Fungal Radiation Resistance Regulatory Networks through the Genome-Wide Transcriptome and Genetic Analyses of *Cryptococcus neoformans*. *mBio* **2016**, *7*, doi:10.1128/mBio.01483-16.
3. Lee, K.T.; So, Y.S.; Yang, D.H.; Jung, K.W.; Choi, J.; Lee, D.G.; Kwon, H.; Jang, J.; Wang, L.L.; Cha, S., et al. Systematic fungal analysis of kinases in the fungal pathogen *Cryptococcus neoformans*. *Nature communications* **2016**, *7*, 12766, doi:doi: 10.1038/ncomms12766.
