# Supplementary material for: Functional Roles of Homologous Recombination and Non-Homologous End Joining in DNA Damage Response and Microevolution in Cryptococcus neoformans
Source: J Fungi (Basel). 2021 Jul 16;7(7):566. doi: 10.3390/jof7070566 (PMC8307084; doi:10.3390/jof7070566)
Supplement: Supplementary file 1 [file jof-07-00566-s001.zip › Table_S2_Jung et al.pdf]

Table S2. Primers used in this study

Primer Name	Sequence (5′—3′)	Comment
B79	TGTGGATGCTGGCGGAGGATA	Screening primer on *ACT1* promter
B1026	GTAAAACGACGGCCAGTGAGC	M13 forward (extended)
B1027	CAGGAAACAGCTATGACCATG	M13 reverse (extended)
B1454	AAGGTGTTCCCCGACGACGAATCG	NSL2
B1455	AACTCCGTCGCGAGCCCCATCAAC	NSR2
B1886	TGGAAGAGATGGATGTGC	NSL-NEO
B1887	ATTGTCTGTTGTGCCCAG	NSR-NEO
B679	CGCCCTTGCTCCTTCTTCTATG	*ACT1* qRT primer 1
B680	GACTCGTCGTATTCGCTCTTCG	*ACT1* qRT primer 2
J1	TGGTGGTGAGGGAAAATG	*RAD51* (CNAG_00720) qRT primer 1
J2	GCACCTCTTCACCATCAAG	*RAD51* (CNAG_00720) qRT primer 2
J1321	GCCACCAACGAGCAAATAG	*KU70* (CNAG_04220) qRT primer 1
J1322	ATGCCAAGGAGGGATTAGG	*KU70* (CNAG_04220) qRT primer 2
J1323	CGGAAGACGCAGAAGGAAG	*KU80* (CNAG_03637) qRT primer 1
J1324	AGGTTCGGTCTTCGTGATG	*KU80* (CNAG_03637) qRT primer 2
J1207	TATCACATCCTGAGCCACG	*KU70* (CNAG_04220) –5′ screening primer for deletion
J1208	AGGGCCAAAGTGCTTTCTG	*KU70* (CNAG_04220) – left flanking primer 1 for deletion
J1209	TCACTGGCCGTCGTTTTACTTGCATGAGTCAACGGAC G	*KU70* (CNAG_04220) – left flanking primer 2 for deletion
J1210	CATGGTCATAGCTGTTTCCTGAAACCATACGGCAGCC ATC	*KU70* (CNAG_04220) – right flanking primer 1 for deletion
J1211	GAATGGAGGTCGGGTGAAC	*KU70* (CNAG_04220) – right flanking primer 2 for deletion
J1212	TTTCCTCTGGCTCCTGAAC	*KU70* (CNAG_04220) – probe primer for Southern blot
J1348	GGTACCgcggccgcAGAAGTATGTCGCGGAATGG	*KU70* (CNAG_04220) – complementation primer L1
J1349	CGTAAATGAGAAGCAGCTTGG	*KU70* (CNAG_04220) – complementation primer R1
J1350	TGGTGGTGGTATGGACATTG	*KU70* (CNAG_04220) – complementation seq 1
J1351	CAGTCGAAACAACCGAACC	*KU70* (CNAG_04220) – complementation seq 2
J1352	CAGGAGCCAGAGGAAAGAG	*KU70* (CNAG_04220) – complementation primer L2
J1353	gcggccgcTCTCCCGTGGTTTATTCCTC	*KU70* (CNAG_04220) – complementation primer R2
J1354	GCCGACGAAGAGGACAAAG	*KU70* (CNAG_04220) – complementation seq 3
J1213	AACGGACATCCAAGCCTC	*KU80* (CNAG_03637) –5′ screening primer for deletion
J1214	AGCCTCGCCAAAGAGTATG	*KU80* (CNAG_03637) – left flanking primer 1 for deletion
J1215	TCACTGGCCGTCGTTTTACCGGCCAACATTCAATGAG C	*KU80* (CNAG_03637) – left flanking primer 2 for deletion
J1216	CATGGTCATAGCTGTTTCCTGTTGCCGAAGACGAACG CAC	*KU80* (CNAG_03637) – right flanking primer 1 for deletion
J1217	TCGTATCCAAGGGCGGTAG	*KU80* (CNAG_03637) – right flanking primer 2 for deletion
J1218	AATGCCCTTACGAGTGTCC	*KU80* (CNAG_03637) – probe primer for Southern blot
J1342	CATATGgcggccgcTGGAAGGCTTCGTACAGG	*KU80* (CNAG_03637) – complementation primer L1
J1343	CATATGAATGATGCTGTTTG	*KU80* (CNAG_03637) – complementation primer R1
J1344	ATGATAGGGCTACGAGTGC	*KU80* (CNAG_03637) – complementation primer seq1
J1345	GGGATGATCTCGGCTGAAG	*KU80* (CNAG_03637) – complementation primer L2
J1346	gcggccgcAGAAAGTACCTTGGCTGCTC	*KU80* (CNAG_03637) – complementation primer R2
J1347	TTCCCATCACTGACCACC	*KU80* (CNAG_03637) – complementation primer seq2
J1271	gcggccgcGCAGCTTGGAGCAGAGTAC	*RAD51* (CNAG_00720)- complementation primer L1
J1272	AGCGTAATCTGGAACATCGTATGGGTACATTGTTCCT GCGAGGTGTTG	*RAD51* (CNAG_00720)- complementation primer R1
J1273	TACCCATACGATGTTCCAGATTACGCTTCGCGAGAGC AGGAGCATGAC	*RAD51* (CNAG_00720)- complementation primer L2
J1274	GGTACCATCTTACATGCTAG	*RAD51* (CNAG_00720)- complementation primer R2
J1275	GGTACCTATGGGATTTACGA	*RAD51* (CNAG_00720)- complementation primer L3
J1276	GGTACCTATGGGATTTACGA	*RAD51* (CNAG_00720)- complementation primer R3
J1567	AGAGCATCGGATAGGCGTG	*URA5* (CNAG_03196) amplification primer L1
J1568	CCAGCCCTATTTCCCAGAC	*URA5* (CNAG_03196) amplification primer R1
J1149	TGCACGTCACGCATCATC	*URA5* (CNAG_03196) sequencing primer 1
J1150	CCATTAGGTCCAGGTGCTC	*URA5* (CNAG_03196) sequencing primer 2
J1623	TGCCATACCATCTCCATACG	*URA3* (CNAG_04961) amplification primer L1

J1624	GTCACGTTGAATGGCAGC	*URA3* (CNAG_04961) amplification primer R1
J1625	TGATTGACGACGGACGGAC	*URA3* (CNAG_04961) sequencing primer 1
J1626	CAATTTGGTGGCGGAGGAG	*URA3* (CNAG_04961) sequencing primer 2
