# Supplementary material for: Functional Roles of Homologous Recombination and Non-Homologous End Joining in DNA Damage Response and Microevolution in Cryptococcus neoformans
Source: J Fungi (Basel). 2021 Jul 16;7(7):566. doi: 10.3390/jof7070566 (PMC8307084; doi:10.3390/jof7070566)
Supplement: Supplementary file 1 [file jof-07-00566-s001.zip › Table_S3_Jung et al.pdf]

Supplementary Table S3. Mutation profiles in the 5-FOA-resistant strains

rad51Δ+RAD51					*rad51Δ*				
	URA3		*URA5*			*URA3*		*URA5*	
clone	DNA mutation[1]	Protein mutation	DNA mutation[1]	Protein mutation	clone	DNA mutation[1]	Protein mutation	DNA mutation[1]	Protein mutation
#1	'ATGGA' insertion	Stop codon change	None	None	#1	'TACGCCTCGC GC' deletion	'YASR(10-13)' deletion	None	None
#2	'GT-AG rule' sequence (G->A) in the 2nd intron region	ND*	None	None	#2	'TACGCCTCGC GC' deletion	'YASR(10-13)' deletion	None	None
#3	'GT-AG rule' sequence (G->A) in the 2nd intron region	ND*	None	None	#3	'TACGCCTCGC GC' deletion	'YASR(10-13)' deletion	None	None
#4	None	None	G546C	E182D	#4	None	None	G124C	A42P
#5	'GT-AG rule' sequence (G->A) in the 2nd intron region	ND*	None	None	#5	'TACGCCTCGC GC' deletion	'YASR(10-13)' deletion	None	None
#6	None	None	G546C	E182D	#6	None	None	C155A	Stop codon change
#7	None	None	C660A	Stop codon change	#7	None	None	G493A	E165K
#8	'GT-AG rule' sequence (G->A) in the 2nd intron region	ND*	None	None	#8	None	None	G484A	D162N
#9	'GT-AG rule' sequence (G->A) in the 2nd intron region	ND*	None	None	#9	None	None	C155A	Stop codon change
#10	'GT-AG rule' sequence (G->A) in the	ND*	None	None	#10	'TACGCCTCGC GC' deletion	'YASR(10-13)' deletion	None	None

	2nd intron region								
#11	‘GT-AG rule’ sequence (G->A) in the 2nd intron region	ND*	None	None	#11	None	None	T185G	L62R
#12	‘GT-AG rule’ sequence (G->A) in the 2nd intron region	ND*	None	None	#12	None	None	G315C	R105S
#13	‘GT-AG rule’ sequence (G->A) in the 2nd intron region	ND*	None	None	#13	‘TACGCCTCGCGC’ deletion	‘YASR(10-13)’ deletion	None	None
#14	‘GT-AG rule’ sequence (G->A) in the 2nd intron region	ND*	None	None	#14	‘TACGCCTCGCGC’ deletion	‘YASR(10-13)’ deletion	None	None
#15	‘GT-AG rule’ sequence (G->A) in the 2nd intron region	ND*	None	None	#15	‘TACGCCTCGCGC’ deletion	‘YASR(10-13)’ deletion	None	None

DNA mutation[1]: a position for nucleotide change in the cDNA sequence

ND[2]: Not determined
